# Supplementary material for: Nanoscale geochemical and geomechanical characterization of organic matter in shale
Source: Nat Commun. 2017 Dec 19;8:2179. doi: 10.1038/s41467-017-02254-0 (PMC5736702; doi:10.1038/s41467-017-02254-0)

Supplementary Table 1. Semi-quantitative ratios derived from IR spectra.

Semi-quantitative index	Index calculation	Band region (cm^{-1})
**Aromaticity**	Aromatic C-H/ Aliphatic C-H_x	$A_{3000-3100}$ / $A_{2800-3000}$
**CH_3/CH_2**	Methyl-to-methylene ratio	A_{2957} / A_{2925}
**C-Factor**	C=O/ (C=O+C=C)<br>oxygenated versus aromatic functional groups	A_{1710} / A_{1710} + $A_{1600-1630}$
**A-Factor**	Aliphatic C-H_x / (Aliphatic C-H_x + Aromatic C=C)	A_{1450} / A_{1450} + $A_{1600-1630}$

Supplementary Figure 1. Solid bitumen reflectance as a function of hydrous pyrolysis temperature.

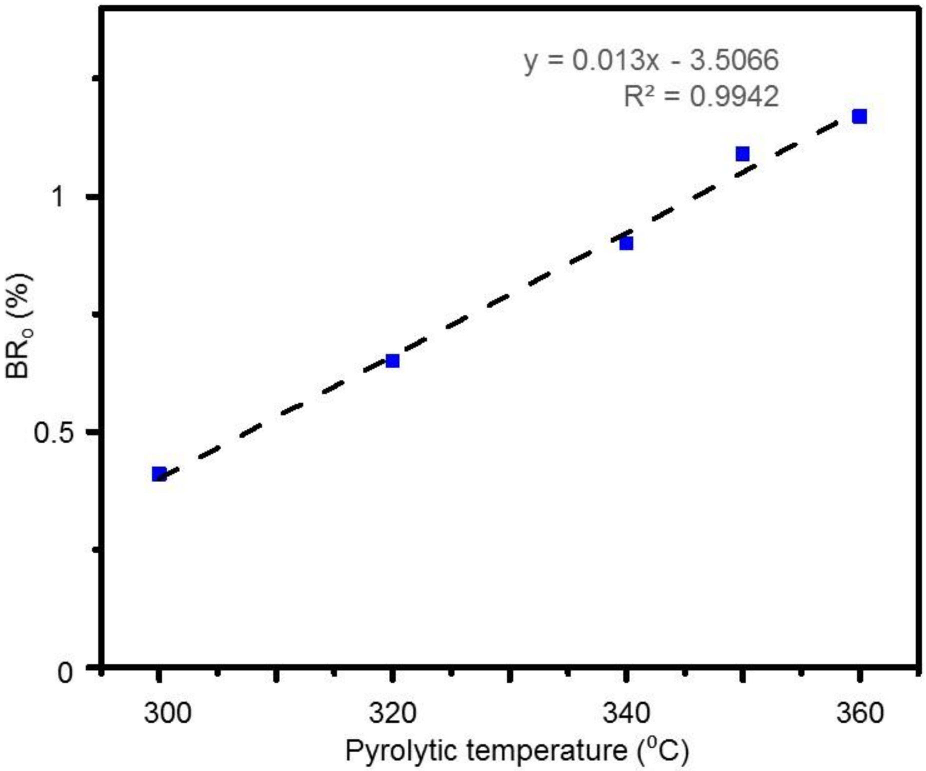

Supplementary Figure 2. Typical localized IR spectra from different locations in Tasmanites, inertinite and solid bitumen particles in the untreated New Albany sample (BR_o = 0.25%)

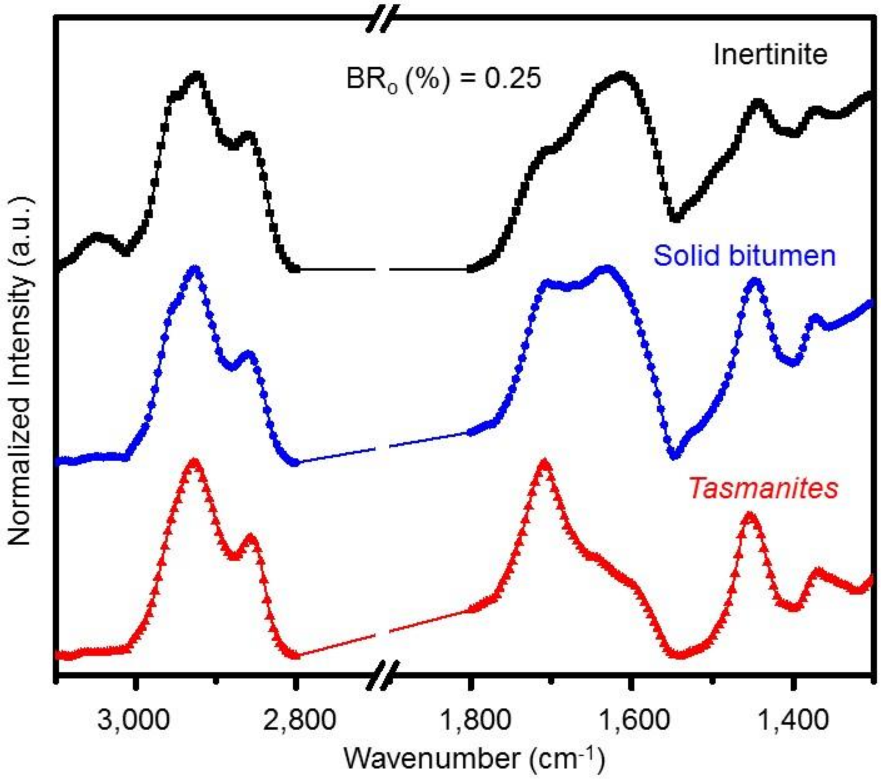

Supplementary Figure 3. Correlative imaging of micron-scale solid bitumen particle. (a) Photomicrograph (white incident light, oil immersion) showing bright solid bitumen embedded in rock matrix of New Albany Shale pyrolyzed under hydrous conditions for 72 hrs at 320°C. (b) Zoom-in to central region of interest via AFM-IR topographic mapping, showing solid bitumen to be lower in relief than mineral. (c) IR absorption mapping at 2,920 cm^{-1}. (d) Mechanical stiffness mapping at 2,920 cm^{-1}.

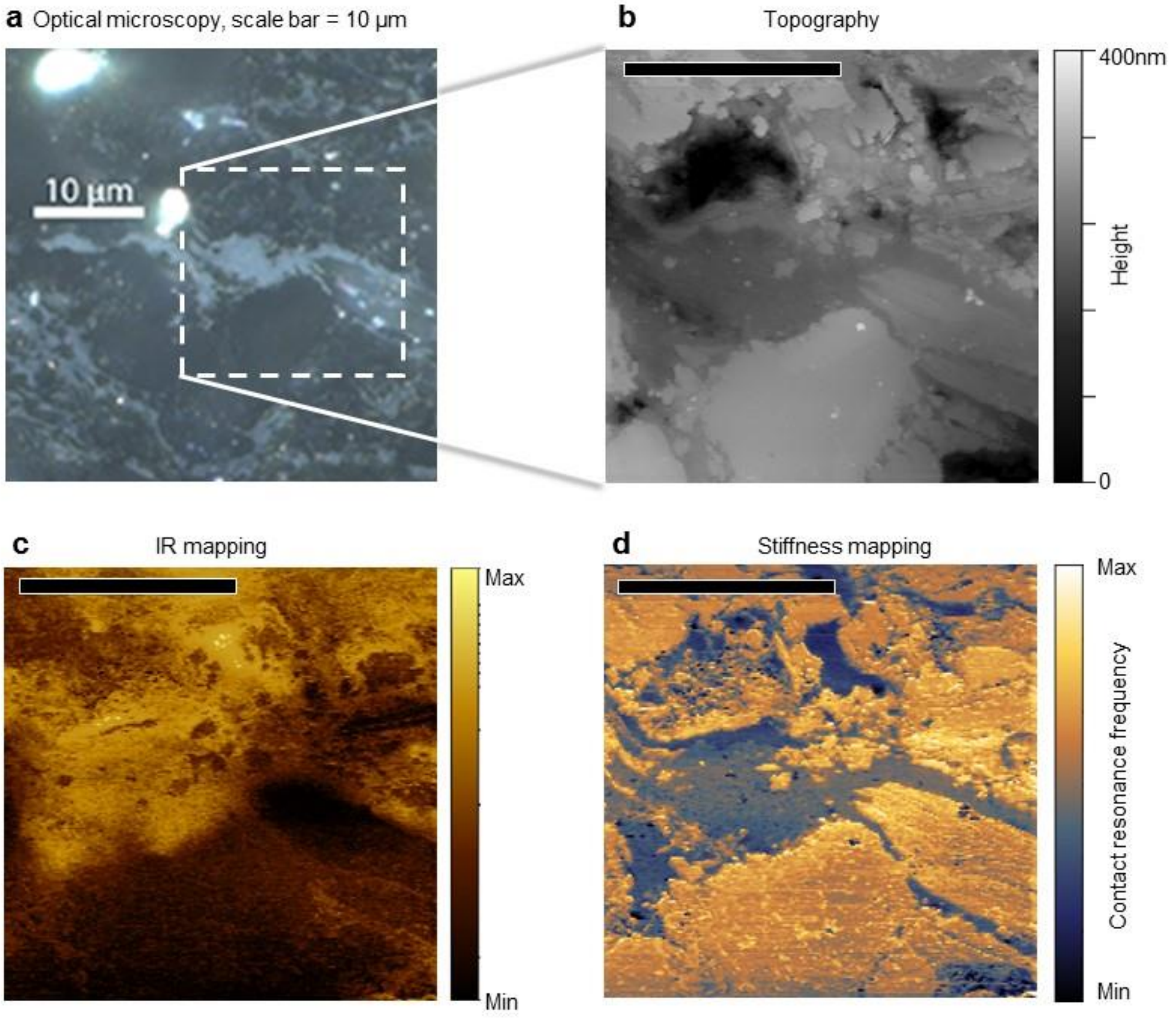

Supplementary Figure 4. Localized IR spectra of different solid bitumen particles in untreated New Albany Shale sample (BR_o = 0.25%). Localized IR spectra recorded and normalized between 1,300-1,800 cm^{-1} and 2,800-3,100 cm^{-1} from localized areas of different solid bitumen particles.

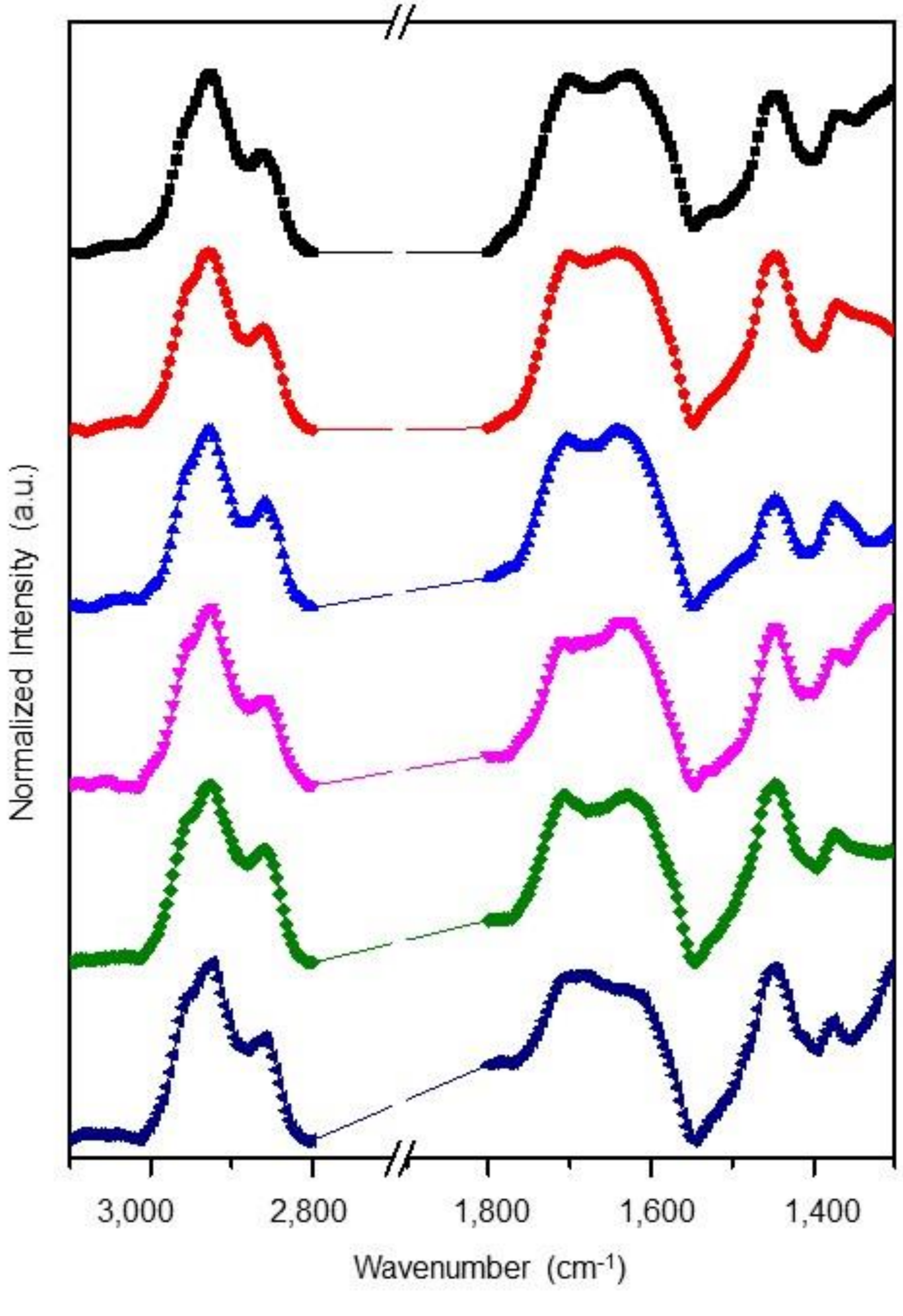

Supplementary Figure 5. Localized IR spectra of different solid bitumen particles in pyrolyzed New Albany Shale sample (BR_o = 0.65%). Localized IR spectra recorded and normalized between 1,300-1,800 cm^{-1} and 2,800-3,100 cm^{-1} from localized areas of different solid bitumen particles.

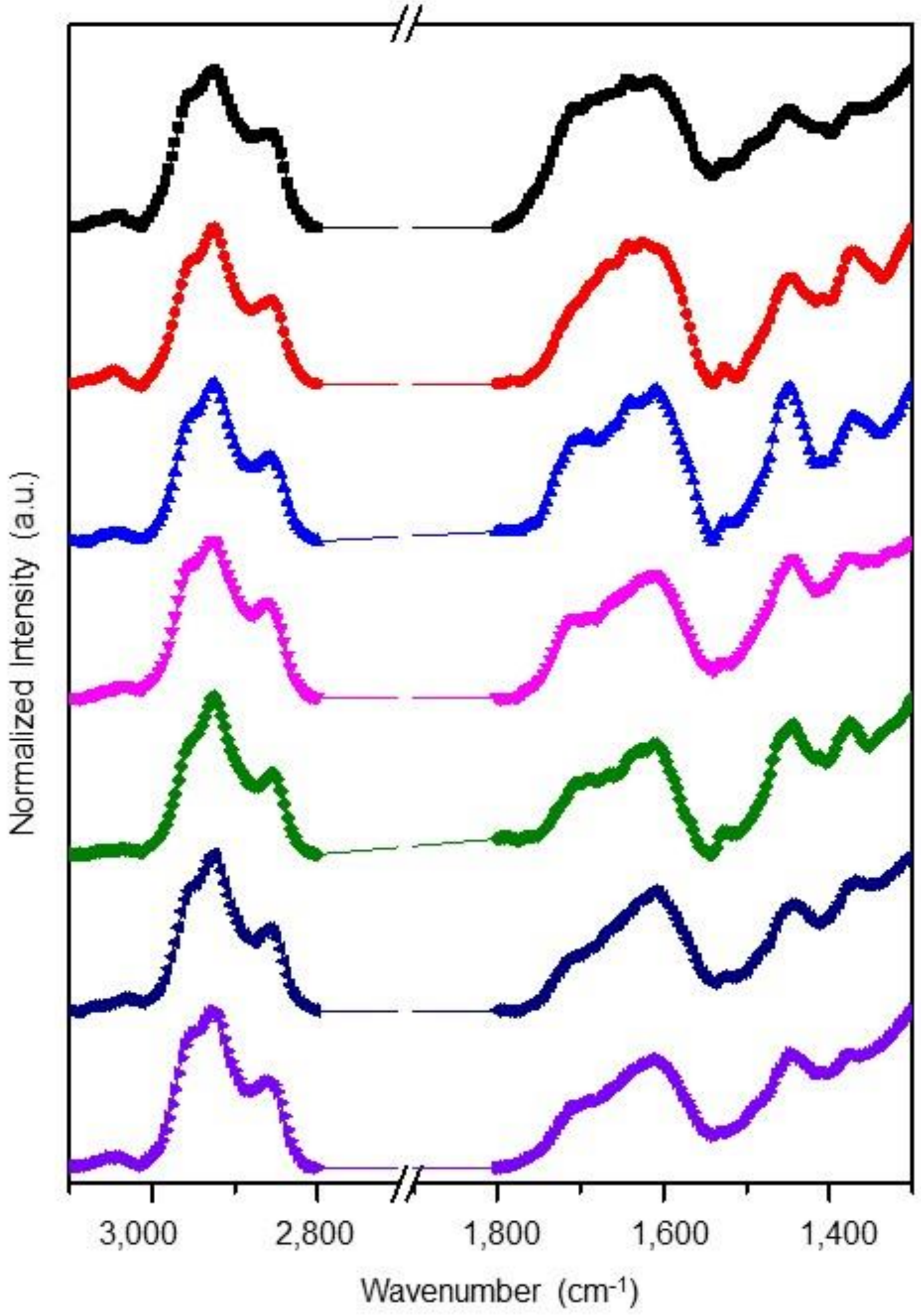

Supplementary Figure 6. Localized IR spectra of different solid bitumen particles in pyrolyzed New Albany Shale sample (BR_o = 0.90%). Localized IR spectra recorded and normalized between 1,300-1,800 cm^{-1} and 2,800-3,100 cm^{-1} from localized areas of different solid bitumen particles.

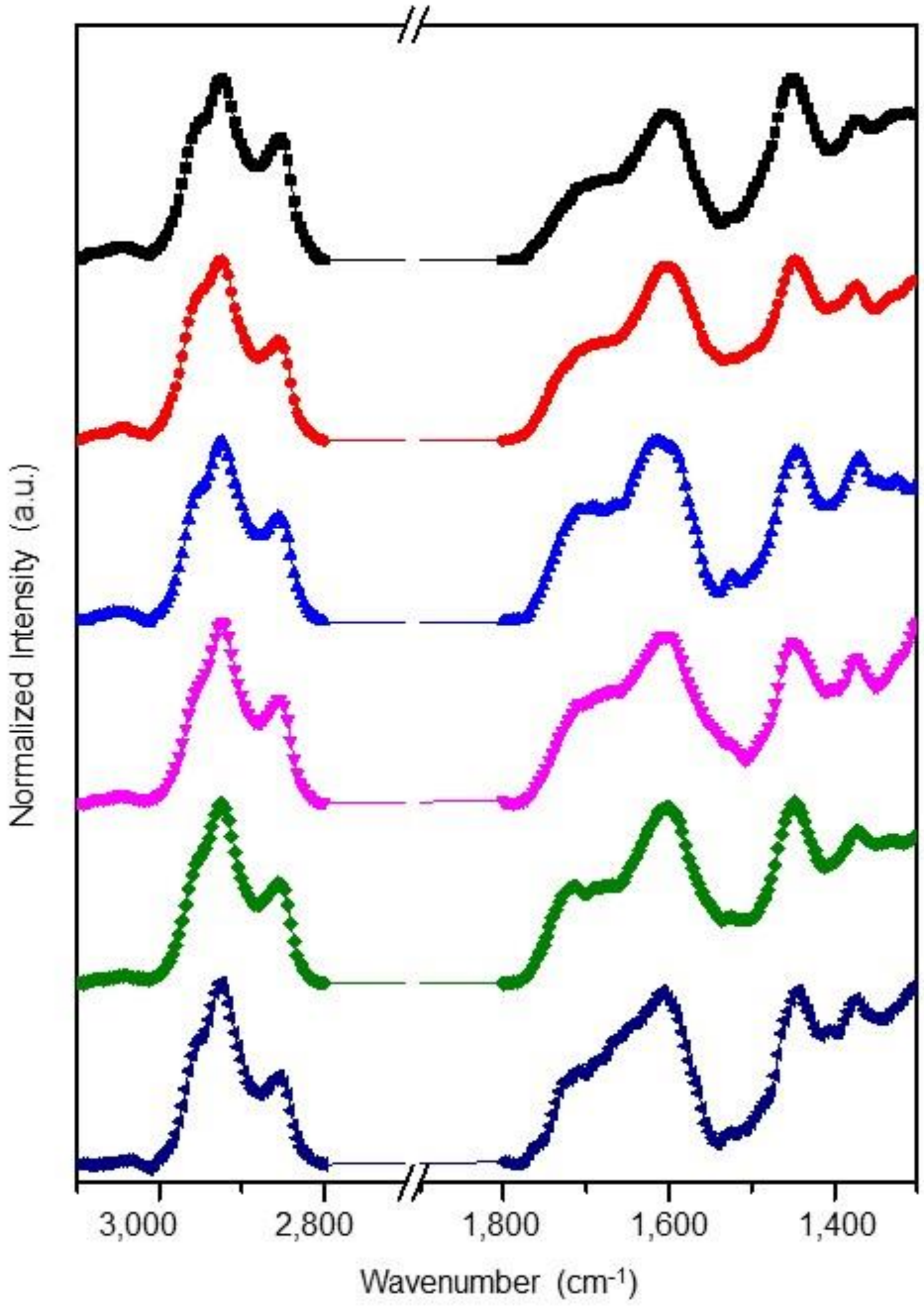

Supplementary Figure 7. Localized IR spectra of different solid bitumen particles in pyrolyzed New Albany Shale sample (BR_o = 1.17%). Localized IR spectra recorded and normalized between 1,300-1,800 cm^{-1} and 2,800-3,100 cm^{-1} from localized areas of different solid bitumen particles.

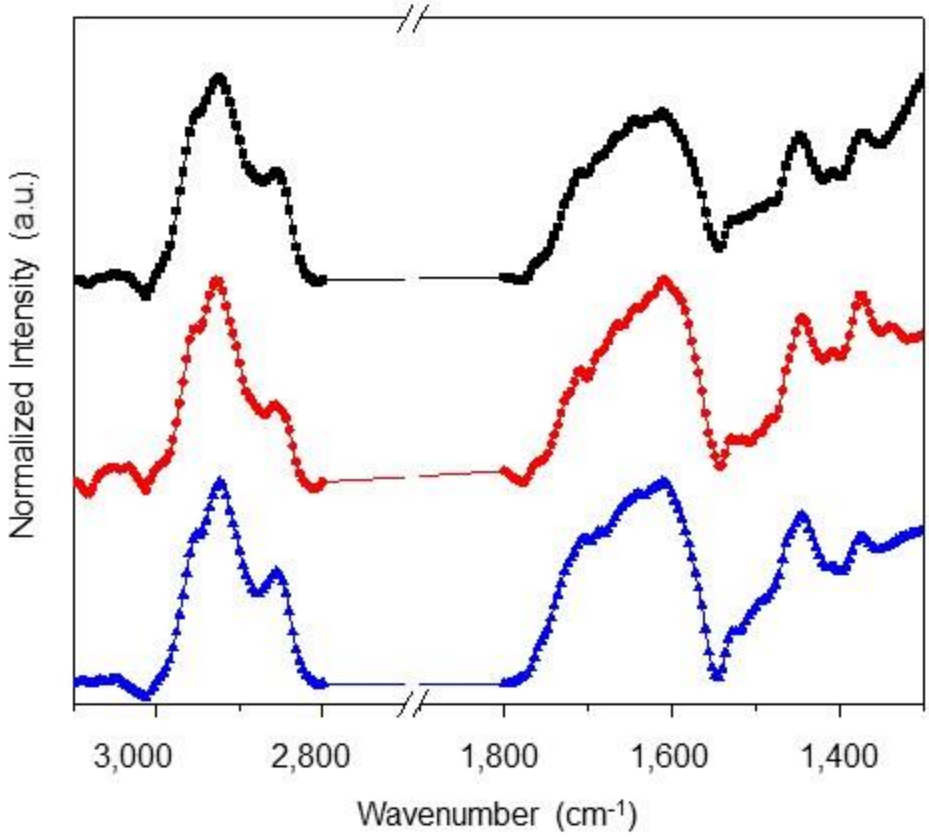

Supplement: Supplementary file 1 — Supplementary Information [file 41467_2017_2254_MOESM1_ESM.pdf]
